# Prevalence of Eating Disorders and Its Associated Risk Factors in Students of a Medical College Hospital in South India

**DOI:** 10.7759/cureus.12926

**Published:** 2021-01-26

**Authors:** Shruti Iyer, Vanishree Shriraam

**Affiliations:** 1 Community Medicine, Sri Ramachandra Institute of Higher Education and Research, Chennai, IND

**Keywords:** eating disorders, medical students, psychological stress, prevalence, screening program

## Abstract

Background and objectives

Eating disorders are some of the most under-researched and difficult to diagnose psychiatric conditions, with a high mortality rate, especially among the adolescent age group. The aim of this study is to determine the prevalence and risk factors for eating disorders among students of a medical college hospital in South India.

Materials and methods

An observational, cross-sectional study was conducted among 332 students of four constituent colleges of a tertiary-care hospital selected by simple random sampling. Their height and weight were recorded. Four major questionnaires were distributed among the students - Demographic details, Eating Attitudes Test (EAT26), Body Shape Questionnaire (BSQ34), and Perceived Stress Scale (PSS). The results were tabulated and analyzed using SPSS software version 16.0 (IBM Corporation, Somers, New York, USA).

Results

The proportion of students who had a high risk for eating disorders was 13%. It was prevalent almost equally in both males and females. High risk for eating disorders was associated with high stress and severe body shape concerns (p<0.001). Other influencing factors were history of counselling, peer pressure, excessive exercise as well as the history of any behavioral symptoms like the use of laxatives and diet pills (p<0.001).

Conclusions

Eating disorder risk is prevalent in a high percentage of medical and paramedical students. High stress and body shape concerns are associated with eating disorders. Only if diagnosed early, with screening programs using questionnaires and further psychiatric evaluation, we can hope to mitigate the complications they incur.

## Introduction

Mental health is an under-recognized field of medicine that has gained traction only in the last decade. A report by the World Health Organization (WHO) revealed that 7.5% of the Indian population suffers from some form of mental disorder. Mental illnesses constitute one-sixth of all health-related disorders and India accounts for nearly 15% of the global mental, neurological, and substance abuse disorder burden [1].

One of the most under-researched topics in India is eating disorders. Eating disorders refer to a group of conditions that involve either insufficient or excessive food intake that is detrimental to an individual's physical and emotional health. Binge eating disorder, bulimia nervosa, and anorexia nervosa are considered to be the most common forms of eating disorders, but in India they present in a less defined manner [2,3].

Eating disorders are extremely serious health issues that affect people of all ages but are mainly seen among adolescents and students [4]. The Multi-Service Eating Disorders Association (MEDA) 4 revealed that nearly 15% of women in the age group of 17 to 24 have eating disorders of some type. Earlier thought to be only a western problem, eating disorders are now seen in adolescents of all racial and socioeconomic groups and more than 75% of these cases begin during adolescence [5].

These are serious psychiatric illnesses with significant morbidity and mortality rates. Eating disorders are predominantly represented by the mental effects of preoccupation with body weight, shape, and diet. There is a multitude of factors that influence these disorders, like socioeconomic status, stress, media, and so on which have not been thoroughly researched [6]. They can also be associated with other psychiatric disorders, like depression and anxiety, making them more harmful and potentially lethal [7,8]. To add to the burden, the diagnosis of eating disorders can be elusive, and more than one-half of all cases go undetected. In India, there is a lack of awareness and a poorly defined diagnostic method for eating disorders.

In such a situation, a thorough screening program is the best strategy for the prevention of serious complications of advanced eating disorders. While eating disorders can only be correctly diagnosed by a trained psychiatrist, regular screening with questionnaires and interviews and further referral to a psychiatrist can potentially aid both the early diagnosis and treatment of these illnesses. Furthermore, increased awareness of its symptoms and presentation among the youth can help in both primary and secondary prevention. The Eating Attitudes Test (EAT26) written by Garner et al. is the most widely used screening tool for eating disorders [9]. Stress is ubiquitous and in a sector like the medical field, it is amplified due to high-pressure situations and is frequently screened using the Perceived Stress Scale (PSS) developed by Cohen [10].

Early diagnosis is the key to reduce the prevalence and complications of these illnesses [11]. There are very few studies concerning eating disorders and their associated risk factors among the Indian population [12-14]. Hence, in this study, we aim to estimate the prevalence of eating disorders and their associated risk factors such as stress, body mass index (BMI), body shape concerns, and other factors among students of various disciplines in a medical college hospital.

## Materials and methods

Study setting

This cross-sectional study was conducted in a multidisciplinary medical college hospital in Chennai. Permission to conduct the study was obtained from the Dean of the institution as well as the heads of the constituent colleges of Nursing, Pharmacy, Allied Health Sciences, and Medicine. The data collection for the study was done from April to June 2019.

Sample size and sampling

The sample size was determined to be 327 students with reference to a similar study in health set up in Karachi in which the eating disorders prevalence among adolescents was found to be 22.7% with a relative precision of 20% of p-value and confidence level of 95% [15]. The list of students from each concerned college was obtained and 80-85 students were selected from the attendance register of each college by simple random sampling using a random number table.

Ethics

Approval to conduct the study was obtained from the Institutional Ethics Committee (Ref No. CSP18/JUN71/185). Written informed consent was obtained from every student after explaining the study and before data collection.

Study subjects

Second- and third-year undergraduate students of the departments of Medicine, Pharmacy, Allied-Health sciences, and Nursing who were willing to participate in the study were included in the study. Students with known psychiatric disorders/those who were then on medication for psychiatric disorders were excluded.

Data collection

The standing height of individuals was measured to ±0.001 m using a wall-mounted stadiometer (Holtain, Fixed Stadiometer, Pembs, UK) and body weight was measured to ±0.1 kg using calibrated electronic scales (Vogel & Halke, SECA Model770, Hamburg, Germany). The BMI was calculated as weight/height^2^ (kg/m^2^) based on the World Health Organisation criteria with the following cutoffs: underweight - BMI under 18.5 kg/m^2^; normal weight - BMI greater than or equal to 18.5 to 24.9 kg/m^2^; overweight - BMI greater than or equal to 25 to 29.9 kg/m^2^; obesity - BMI greater than or equal to 30 kg/m^2^ [16].

The students of each constituent college were assembled and they then filled a pretested, piloted, structured self-administered questionnaire containing background information and three validated questionnaires for eating disorders risk (EAT26), stress (PSS), and body shape concern (BSQ). The investigator explained every question as they filled it to ensure that there were no misconceptions about the meaning of any question as well as clarifying doubts if any.

Questionnaires

Eating Disorders - EAT26

The questionnaire addresses three main aspects of these disorders and has three sub-scales to test - dieting, food preoccupation, and oral control. The EAT26 is the first step in the screening process for eating disorders [9]. There are 26 questions and they are scored as follows - Always - 3, Usually - 2, Often - 1, Sometimes, Rarely, Never at 0. (Question 26 is scored in reverse.)

A score of 20 or more on the EAT26 is considered to be a good determinant of high risk for an eating disorder, while a score less than 20 is low risk. The EAT26 is the most widely used screening measure to determine if people have an eating disorder that needs professional attention. It also assesses five symptoms that are strong predictors for eating disorder risk.

Stress - PSS

The PSS is a comprehensive short questionnaire to measure the frequency of situations in a person's life where they feel helpless and out of control [10]. It is the most widely used psychological instrument for measuring the perception of stress. The questions in the PSS ask about thoughts and experiences over the preceding month.

A score of 27 to 40 in the PSS is considered to be high stress, 13 to 27 is moderate stress, and less than 13 is low stress.

Body Dissatisfaction - BSQ 34

The Body Shape Questionnaire (BSQ) is a self-reported validated questionnaire that was developed to measure concerns about body shape that was constructed by Cooper et al. in 1987 [17]. It analyses the discomfort of various aspects of body shape based on thoughts and feelings experienced over the past four weeks. The maximum score is 204, and a higher score indicates more dissatisfaction and discomfort with one's body.

A score greater than 140 raises a severe concern with body shape, 111 to 140 elicits moderate concern, 80 to 111 - a mild concern, and <80 is no concern.

Statistical analysis

Data entry and analysis of the variables was done using Statistical Package for Social Sciences (SPSS) version 16 (IBM Corporation, Somers, New York, USA) software. Chi-square test was done as a test of significance to find the difference in proportions and Student’s t-test to find the difference in means between the groups. Pearson Correlation coefficient was calculated to look for a correlation between two quantitative variables. P-value <0.05 was considered as statistically significant.

## Results

A total of 332 students participated in the study. The frequencies were almost equally distributed across all the four constituent colleges. All the participants were aged between 18 and 21 years. Of the respondents, 43.67% were male while 56.33% were female, as seen in Table 1. Close to half of the students were in hostel and homes.

**Table 1:** Prevalence of Eating Disorders Among Students With Different Background Characteristics: (N=332)

	N (%)	High Risk (≥20) N (%)	Low Risk (<20) N (%)
Course			
Nursing	80 (24.1)	9 (11.25)	71 (88.75)
Pharmacy	91 (27.4)	11 (12.09)	80 (87.90)
Allied health sciences	80 (24.1)	11 (13.75)	69 (86.25)
MBBS	81 (24.4)	12 (14.81)	69 (85.18)
Age (years)			
18	71 (21.3)	6 (8.45)	65 (92.55)
19	94 (28.3)	16 (17.02)	78 (82.99)
20	70 (21)	11 (15.71)	59 (84.28)
21	97 (29.2)	10 (10.31)	87 (89.69)
Gender			
Male	145 (43.67)	18 (12.4)	127 (87.6)
Female	187 (56.33)	25 (13.4)	162 (86.6)
BMI			
<18.5 - underweight	63 (19)	7 (13.20)	46 (86.79)
18.5 - <25 Normal	183 (55.1)	21 (13.37)	136 (86.62)
25 - <30 Overweight	71 (21.4)	14 (19.44)	58 (80.55)
>30 - Obese	13 (3.9)	1 (7.7%)	12 (92.3)
Place of stay			
Hostel	155 (46.7)	22 (14.19)	133 (85.81)
Home	173 (52.1)	19 (10.98)	154 (90.02)
Apartment	4 (1.2)	2 (50)	2 (50)
Exercise			
Everyday	145 (43.7)	24 (22.22)	84 (87.78)
3 days per week	80 (24.1)	6 (6)	94 (94)
1-3 days per week	53 (16)	4 (7.54)	49 (92.46)
Never	54 (16.3)	9 (17.64)	42 (82.36)
History of counselling			
Yes	79 (23.8)	18 (22.78)	61 (77.21)
No	253 (76.2)	25 (9.88)	228 (90.11)
Difficulty with peers			
Never had problems	62 (18.7)	5 (8.06)	57 (91.93)
Rarely	116 (34.9)	10 (8.62)	106 (91.37)
Sometimes	128 (38.6)	5 (4.76)	105 (95.23)
Usually	26 (7.8)	23 (52.27)	21 (47.72)

Of all the participants, 23.8% reported that they had attended counseling in the past (but were not currently in counseling; Table 1) from therapists for various disorders like stress, anxiety, or depression. None of the participants reported being counseled for eating disorders. When questioned about difficulties they experienced with peers, they were asked to answer on the basis of how often they felt pressurized to act a certain way, to engage in behavior they did not feel comfortable with or were bullied in any form. Only 7.8% of the population reported having regular difficulties with their peers (Table 1).

The term ‘eating disorders’ was familiar to 62% of the population, but they did not know what it included, its symptoms, presentations and had never encountered anyone who had suffered from the same. About 10.5% of the students in a health facility had never heard of the term 'eating disorder’ and had no knowledge about it. A total of 241 students (72.5%) in a medical university revealed that they did not know how to recognize an eating disorder or whom to approach for help for the same (not shown in the table).

**Figure 1:**
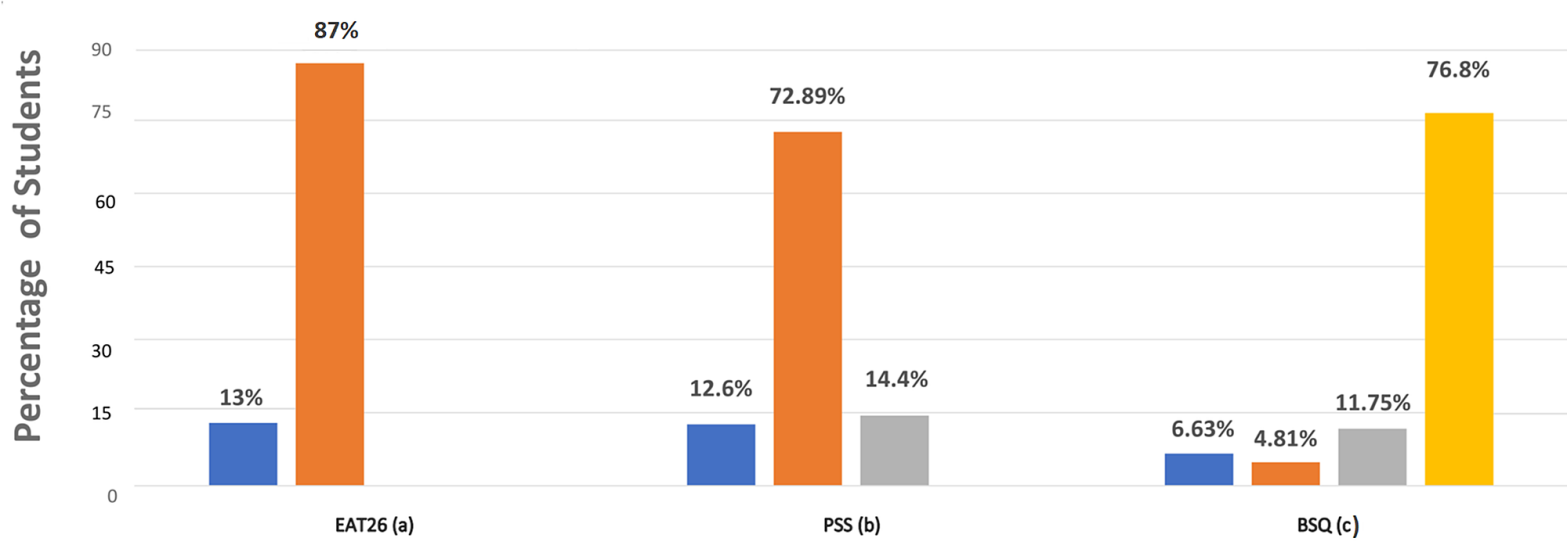
Proportion of Students in Different Risk Categories in the Three Questionnaires (a) High risk (13%) and low risk (87%); (b) high stress (12.6%), moderate stress (72.8%), and low stress (14.4%); (c) severe concern (6.63%), moderate concern (4.8%), mild concern (11.75%), and no concern (76.8%).

The EAT26 results revealed that 43 (13%) of the participants scored above 20 on the test putting them at high risk for a possible eating disorder (Figure 1). Among males, 12.4% reported high-risk scores while 13.4% of the females had high-risk scores (Table 1). The BMI was also assessed and cross-tabulated with the risk of eating disorders and was not found to be statistically significant (p=0.08). People with extreme exercise schedules - daily rigorous exercise or no exercise had a higher EAT26 score (p=0.04*) than those with regular schedules.

The individual EAT26 behavioral symptom prevalence was also analyzed and cross-tabulated with the EAT26 score (Table 2). It is seen that 82.64% of those with one (or more) symptom had a high EAT26 score (p=0.004)*.

**Table 2:** Behavioral Symptoms and Its Association With EAT26 Scores

Symptom	N (%)	High Risk (≥20) N (%)	Low Risk (<20) N (%)
H/o eating binges	61 (18.4)	33(54.09)	28 (45.9)
H/o self-induced vomiting	17 (5.1)	16 (94.11)	1 (5.88)
H/o use of diet pills/laxatives	8 (2.4)	6 (75.00)	2 (25.00)
H/o excessive exercise to reduce weight	27 (8.1)	24 (88.89)	3 (11.10)
H/o loss of weight >10 kg in 6 months	18 (5.4)	11 (61.11)	7 (38.88)

**Figure 2:**
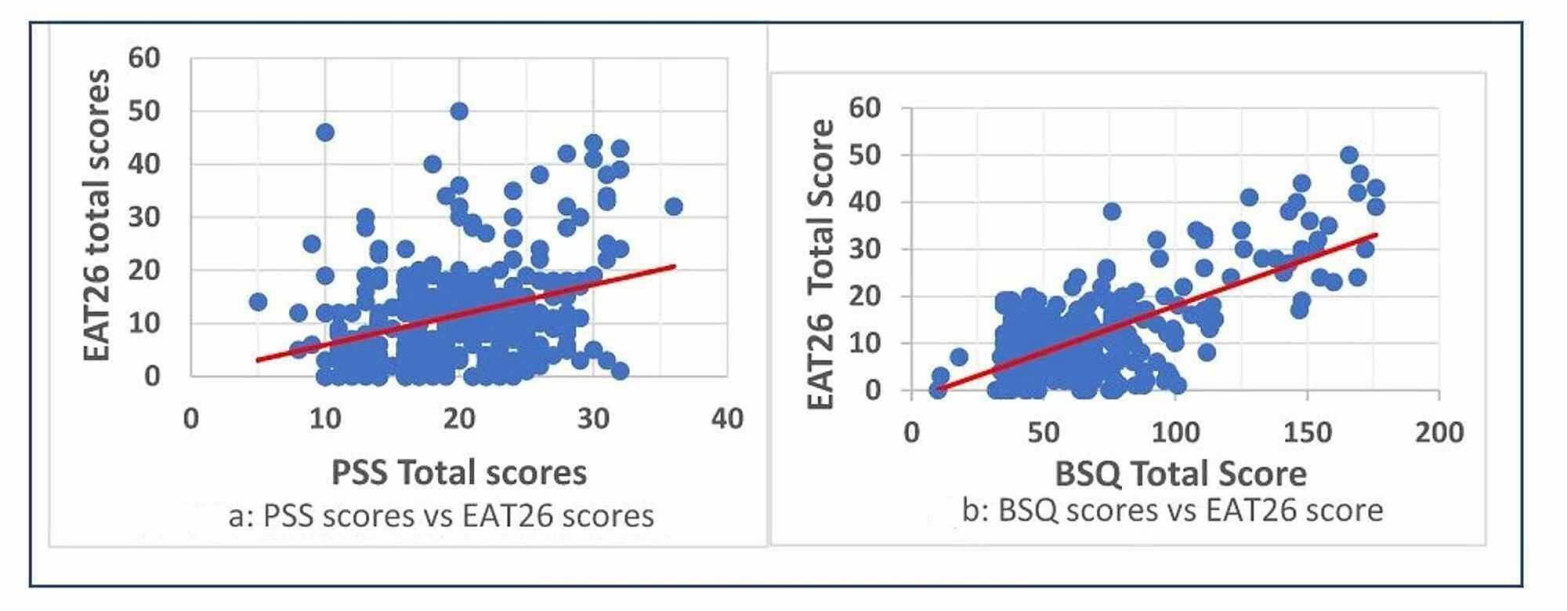
(a) Correlation Between PSS Scores and EAT26 Scores (Pearson Coefficient 0.385, P<0.001) and (b) Correlation Between BSQ34 Scores and EAT26 Scores (Pearson Coefficient 0.683, P<0.001)

The PSS scale revealed that 42 students (12.6%) are under high stress with a score of 27 and above (Figure 1). When the association between stress and eating disorder was analyzed 79.1% (n=34) of the eating disorder high-risk students fell in the high-stress category, 18.6% (n=8) were in the moderate stress category and 2.3% (n=1) were in the low-stress category (p<0.001*; not shown in the table). The Pearson correlation coefficient between PSS scores and EAT26 was found to be 0.385 which indicates a moderate correlation and this was found to be statistically significant (p<0.001*; Figure 2a).

**Figure 3:**
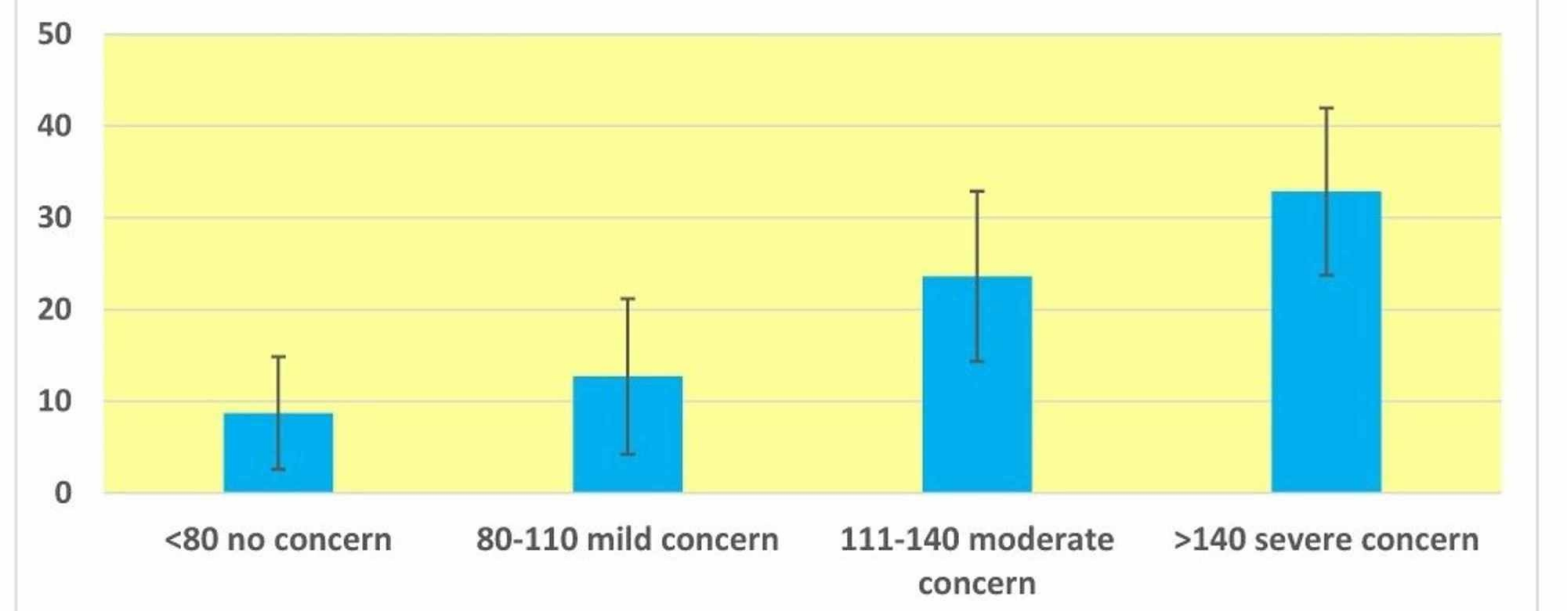
Error Bar Diagram Showing Mean (SD) Score of EAT26 at Different Levels of Body Shape Concerns

From the analysis of the BSQ questionnaire, it is found that 6.6% and 4.8% of the students were having severe and moderate body shape concerns, respectively (Figure 1). As the severity of body shape concern increased the mean (SD) score of the EAT26 also increased (Figure 3). Upon cross-tabulation, 20 of the 22 (90.9%) people who showed severe concern on the BSQ scale also had high risk on the EAT26 scale (p<0.001)* (not shown in the table). The correlation between EAT26 scores and BSQ was high (the Pearson correlation coefficient 0.683) and was statistically significant (p<0.001; Figure 2b).

## Discussion

This study establishes the prevalence of eating disorders among the students of the health sector to be 13%. Although this is lower than some studies in a different demographic, it is still a significant proportion [18]. In the first medical college study in 1995, by Srinivasan et al., 14.8% of the participants were deemed to be at risk for eating disorders using EAT26 and BITE questionnaires. Surprisingly, this study revealed that a majority of eating disorders in India did not fit under the umbrella of anorexia nervosa or bulimia nervosa, but presented as eating disorder of no specific type (EDNOS/EDS) [14]. Since then many studies have shown an increased prevalence among university students. In a study conducted among 435 medical students in Karachi, 22.75% of individuals were found to be at high risk of eating disorders, and in another study in Bangladesh, using EAT26 questionnaire, it was 37.6%.

The prevalence was almost equal in males and females, which is a rare finding, as in most demographics the male prevalence is lower [19]. Our study shows that males are also at significant risk for eating disorders and hence must be thoroughly screened. Risk factors that showed a significant relationship with eating disorders include exercise, stress, past history of psychiatric illnesses, peer pressure, and so on. The prevalence was higher in those on the extremes, that is those who exercised daily or never exercised. Since both anorexia and bulimia are driven by a desire to be thin, excessive exercise can also be a red flag, as it indicates compensatory behavior.

This study shows that only a quarter of the students in the institution (27.4%) were aware of the presenting symptoms and prognosis of eating disorders whereas a majority were not. This is an alarming situation as it implies that non-healthcare associated students might have even lesser knowledge. This could lead to many missing diagnoses, which might also underestimate the national prevalence. If diagnosed early, many can be managed with cognitive behavioral therapy [20].

A past history of counseling, for causes other than eating disorders, inclusive of a therapist/psychiatrist session also showed a significant association with EAT26 scores, which leads to the hypothesis that a history of anxiety, depression, or other mental illnesses can increase the risk of an eating disorder. Our study did not report a significant correlation between BMI and eating disorders, unlike some others. However, this can be explained by the fact that bulimia nervosa, eating disorder of no specific form (EDNOS), Binge eating disorder all present with normal or even elevated BMI [21,22]. Students who reported a regular difficulty with peers also had a higher EAT26 score, suggesting that interpersonal relationships also play a role in this complicated pathology [23].

The correlation between stress and eating disorders was found to be significant leading to the hypothesis that high stress is associated with a greater risk for psychiatric disorders like eating disorders. High-stress environments also lead to consumption of a poor diet, due to paucity of time and knowledge which could be the inciting factor for a more serious illness [24].

The results of the BSQ also showed a high correlation coefficient with the EAT26 Scores which leads to the hypothesis, that body dissatisfaction is a red alert factor for a potential eating disorder [25]. Balhara and Mathur in 2012 revealed similar findings and pointed out that since body image is highly volatile among adolescents, this puts them in an even more vulnerable position to fall prey to an eating disorder. Both BSQ and EAT26 have been used as screening devices by experienced psychiatrists as well and it is imperative that we administer these to all adolescents, since early diagnosis and treatment are the keys to prevent complications [26].

This study sheds new light on the risk factors of eating disorders and also reports a significant association between body shape concerns, stress, and eating disorders, which has not been assessed in the Indian population [27]. But one limitation of the study is that this is done in a single institution which might be limiting the generalizability. Also, the diagnosis of eating disorders has not been confirmed by psychiatrists which would give the true prevalence of the conditions whereas this study gives the prevalence of those at high risk for eating disorders by a screening test.

It is essential that the awareness of eating disorders - what they are, presenting symptoms, and treatment is spread among all populations with a focus on adolescents and young adults. Those who report signs of high stress must also be carefully assessed for other psychiatric disorders. Early diagnosis via screening questionnaires and possible symptoms could be the solution to reducing the prevalence of these disorders. It should be universally adopted among all universities to screen for eating disorders [28,29]. Further studies are needed to establish a clear causal relationship between the risk factors and eating disorders [30].

## Conclusions

The prevalence of eating disorders risk is high among medical and paramedical students, but the awareness is low. High stress, body shape concerns, and behavioral symptoms are significantly associated with eating disorders. Routine screening with referral services in every institute will go a long way in preventing these serious psychiatric illnesses.
